# The synergistic action of accessory enzymes enhances the hydrolytic potential of a “cellulase mixture” but is highly substrate specific

**DOI:** 10.1186/1754-6834-6-112

**Published:** 2013-08-03

**Authors:** Jinguang Hu, Valdeir Arantes, Amadeus Pribowo, Jack N Saddler

**Affiliations:** 1 Forestry Products Biotechnology/Bioenergy Group, Wood Science Department, University of British Columbia, 2424 Main Mall, Vancouver, BC V6T 1Z4, Canada

## Abstract

**Background:**

Currently, the amount of protein/enzyme required to achieve effective cellulose hydrolysis is still too high. One way to reduce the amount of protein/enzyme required is to formulate a more efficient enzyme cocktail by adding so-called accessory enzymes such as xylanase, lytic polysaccharide monooxygenase (AA9, formerly known as GH61), etc., to the cellulase mixture. Previous work has shown the strong synergism that can occur between cellulase and xylanase mixtures during the hydrolysis of steam pretreated corn stover, requiring lower protein loading to achieve effective hydrolysis. However, relatively high loadings of xylanases were required. When family 10 and 11 endo-xylanases and family 5 xyloglucanase were supplemented to a commercial cellulase mixture varying degrees of improved hydrolysis over a range of pretreated, lignocellulosic substrates were observed.

**Results:**

The potential synergistic interactions between cellulase monocomponents and hemicellulases from family 10 and 11 endo-xylanases (GH10 EX and GH11 EX) and family 5 xyloglucanase (GH5 XG), during hydrolysis of various steam pretreated lignocellulosic substrates, were assessed. It was apparent that the hydrolytic activity of cellulase monocomponents was enhanced by the addition of accessory enzymes although the “boosting” effect was highly substrate specific. The GH10 EX and GH5 XG both exhibited broad substrate specificity and showed strong synergistic interaction with the cellulases when added individually. The GH10 EX was more effective on steam pretreated agriculture residues and hardwood substrates whereas GH5 XG addition was more effective on softwood substrates. The synergistic interaction between GH10 EX and GH5 XG when added together further enhanced the hydrolytic activity of the cellulase enzymes over a range of pretreated lignocellulosic substrates. GH10 EX addition could also stimulate further cellulose hydrolysis when added to the hydrolysis reactions when the rate of hydrolysis had levelled off.

**Conclusions:**

Endo-xylanases and xyloglucanases interacted synergistically with cellulases to improve the hydrolysis of a range of pretreated lignocellulosic substrates. However, the extent of improved hydrolysis was highly substrate dependent. It appears that those accessory enzymes, such as GH10 EX and GH5 XG, with broader substrate specificities promoted the greatest improvements in the hydrolytic performance of the cellulase mixture on all of the pretreated biomass substrates.

## Background

Achieving good sugar yields from pretreated lignocellulosic substrates when using low enzyme loadings continues to be a challenge for biochemically based biomass-to-biofuels processes [1,2]. One approach that various groups have assessed is to formulate more efficient enzyme cocktails by adding so-called accessory enzymes, such as hemicellulases and lytic polysaccharide monooxygenase (AA9, formerly known as GH61) [3-5], and/or non-hydrolytic, chain separating proteins such as swollenin and expansin [6-8] to the enzyme mixture. Recent work has shown that, when family 11 endo-xylanases were added to commercial cellulase preparations, their synergistic cooperation not only substantially enhanced the hydrolysis extent of both the glucan and xylan present in steam pretreated corn stover, it also dramatically reduced the required cellulase dosage (about 7 times) needed to achieve reasonable cellulose hydrolysis yields (>70%) [3]. Even though higher amount of xylanase was still required to achieve better hydrolysis, the overall total protein loading either did not change or was slightly lower.

Based on their amino acid sequence similarities, most xylan hydrolyzing enzymes belong to either glycoside hydrolase family 10 (GH10) or family 11 (GH11) [9-11]. Family 10 endo-xylanases (GH10 EX) have been shown to predominantly attack glycosidic linkages next to a substituted xylose residue, requiring at least two unsubstituted xylose residues and it tends to exhibit broad catalytic versatility [9,12]. In contrast, family 11 endo-xylanases (GH11 EX) require at least three consecutive unsubstituted xylose residues. GH11 EX cannot cleave glycosidic linkages next to a branch [10,13], and they are sometimes characterized as “true” xylanases because of their exclusive activity on D-xylose containing substrates [14].

Although there is a considerable amount of information known about the differences between these two major families of endo-xylanases regarding their structure, catalytic mechanisms and specific activities on “model” xylanolytic substrates [12,15,16], there has been little work carried out regarding their hydrolytic potential or their interactions with cellulases during hydrolysis of industrially relevant pretreated lignocellulosic substrates [15-17]. However, previous work has shown that GH10 EX addition was more effective on corn fibre and hydrothermally pretreated wheat straw [16,17] while GH11 EX was more hydrolytic on destarched wheat bran arabinoxylan [15]. Although synergistic cooperation between GH10 EX and GH11 EX was observed [16,18] during the hydrolysis of corn fiber, a similar level of synergism was not observed [15] during hydrolysis of arabinoxylan obtained from wheat bran. A related study [19] showed that the addition of both GH10 EX and GH11 EX along with other enzymes resulted in a more effective enzyme cocktail when hydrolysing the cellulosic component of ammonia fiber expansion (AFEX) pretreated corn stover. This earlier work suggested that GH10 EX had broad catalytic specificity and that this enzyme might prove to be a better candidate for enhancing the hydrolysis of more realistic biomass substrates.

Xyloglucanase addition has also been shown to enhance the hydrolyzability of various lignocellulosic substrates when added to a cellulase mixture [20,21]. Xyloglucanase is known to hydrolyze xyloglucan, which is one of the major components of the primary cell wall of higher plants and it has been shown to limit the accessibility of cellulase enzymes to the cellulosic component [20,22-24]. Benko et al., [20] showed that a xyloglucanase from *Trichoderma reesei* enhanced the catalytic performance of a cellulase mixture during hydrolysis of several lignocellulosic substrates. Related work also showed that the depolymerization and re-arrangement of the linkages in xyloglucan by hydrolases or transferases was an essential step in plant cell wall expansion and deposition during cell growth [25]. This suggested that the cleavage of glycosidic linkages within the xyloglucan resulted in the swelling of the cellulose microfibrils [22,24] which has been shown to increase cellulose accessibility and, consequently, effectiveness of enzyme hydrolysis [22].

The work reported here has looked at the potential synergistic interaction between cellulases and several hemicellulosic hydrolyzing enzymes (GH11 EX, GH10 EX), and a family 5 xyloglucanase (GH5 XG) during the enzymatic hydrolysis of various steam pretreated lignocellulosic substrates. As well as adjusting the “cocktails” at the beginning on the hydrolysis we also tried to “re-start” hydrolysis by the addition of further enzymes when the rate of hydrolysis had leveled off. As is explained later, although GH11 EX exhibited higher activity on “model” xylanolytic substrates, GH10 EX addition was better able to boost the hydrolytic potential of the cellulase monocomponents during hydrolysis of pretreated lignocellulosic substrates. However, the observed improvements in hydrolysis yields were highly substrate dependent.

## Results

### Chemical composition of pretreated substrates

Of the three pretreated agricultural residues (corn stover, sweet sorghum bagasse, and corn fiber), the steam pretreated sorghum bagasse (SPSB) and the steam pretreated corn stover (SPCS) showed only slight differences in their xylan content (9.8% and 7.0%, respectively) (Table 1). As expected, the steam pretreated corn fiber (SPCF) had a much higher xylan content (15.3%) with the highly branched xylan backbone resulting in a significant amount of arabinosyl (6.9%), galactosyl (2.8%), and mannosyl (2.2%) residues as compared to the SPCS and SPSB substrates (Table 1). When the poplar chips were steam pretreated at increasing severities, the cellulosic rich, water insoluble fractions had a xylan content of 6.6% (SP180) and 3.7% (SP200) respectively. These substrates were subsequently used to try to assess how the relative amount of residual hemicellulose might influence the benefit of adding accessory enzymes, such as xylanases, on the overall ease of cellulose hydrolysis. The steam pretreated lodgepole pine (SPLP) contained undetectable levels of mannan, typically found in softwood hemicelluloses (Table 1).

**Table 1:** Steam pretreatment conditions and chemical composition of pretreated lignocellulosic substrates

**Substrate**	**Pretreatment conditions**	**Composition of pretreated feedstocks**						**Abbreviation**
		***Ara***	***Gal***	***Glu***	***Xyl***	***Man***	***AIL***	
Corn fiber	190°C, 5 min, 3% SO_2_	6.9	2.8	38.2	15.3	2.2	12.6	SPCF
Sweet sorghum bagasse	190°C, 5 min, 3% SO_2_	0.6	0.8	54.3	9.8	1.0	25.8	SPSB
Corn stover	190°C, 5 min, 3% SO_2_	1.0	0.7	56.1	7.0	1.1	27.0	SPCS
Poplar	180°C, 5 min, 4% SO_2_	0.3	0.8	59.8	6.6	1.3	30.4	SPP180
	200°C, 5 min, 4% SO_2_	0.3	0.8	59.3	3.7	1.2	33.9	SPP200
Lodgepole pine	200°C, 5 min, 4% SO_2_	bdl	bdl	46.4	bdl	bdl	45.0	SPLP

*Ara* Arabinan, *Xyl* Xylan, *Glu* Glucan, *Gal* Galactan, *Man* Mannan, *AIL* Acid Insoluble Lignin, *bdl* below detectable level.

### Determination of enzymatic activities using “model” substrates

The xylanase, xyloglucanase, endo-glucanase, β-glucosidase and β-xylosidase activities of the purified enzymes were determined as detailed in the material and methods section (Table 2). As expected, both family endo-xylanases were able to effectively hydrolyze all of the xylan “model” substrates, while the other enzymes showed very low or undetectable levels of activity. The GH11 EX showed substantially higher hydrolytic activity (190–230 U/mg) on all of the xylan substrates than did GH10 EX (100–160 U/mg). This difference in activity was also observed previously [26] when using thermostable recombinant xylanases from *Nonomuraea flexuosa* and *Thermoascus aurantiacus*. When various *p*-nitrophenyl (pNP) substrates were used to assess any differences between the two xylanases, the GH10 EX showed detectable activities on pNPC, pNPG, and pNPX, suggesting that GH10 EX had a broader catalytic versatility and may also have higher hydrolysis efficiency towards short xylooligomers (p-NPX activity) as compared to GH11 EX. In contrast, the family 5 xyloglucanase (GH5 XG) was the only enzyme that showed significant activity towards xyloglucan (146 U/mg) (Table 2). It also displayed noticeable hydrolytic activity towards CMC (3.7 U/mg). Of the various cellulase monocomponents that were assessed, only Cel7A showed any activity on the p-NPC substrate and it also showed detectable levels of activity on other “model” substrates such as CMC, xylan (birch wood, beech wood, oat spelts) and xyloglucan (Table 2). As expected, β-glucosidase was the only enzyme that displayed notable activity on the p-NPG substrate (0.4 U/mg).

**Table 2:** Specific activities (U/mg) of the purified enzymes assessed on “model” substrates

**Enzymes**	**Birch wood xylan**	**Beech wood xylan**	**Oat spelts xylan**	**Xyloglucan**	**CMC**	**p-NPC**	**p-NPG**	**p-NPX**
GH11 EX	193.2	191.5	229.3	n/a	n/a	n/a	n/a	n/a
GH10 EX	103.6	119.2	162.9	n/a	n/a	<0.2	<0.1	0.5
GH5 XG	n/a	n/a	n/a	145.6	3.7	n/a	n/a	n/a
Cel7A	<0.2	0.2	<0.2	1.24	<0.2	0.3	n/a	n/a
GH3 BG	n/a	0.2	0.35	n/a	n/a	<0.1	0.4	n/a

n/a - negligible activity was detected.

### Interaction of GH11 EX with cellulase monocomponents

Earlier work had shown the strong synergistic cooperation between a commercial cellulase mixture (Novozymes Celluclast 1.5 L) and a commercial xylanase mixture (Genencor Multifect Xylanase) during hydrolysis of SPCS [3]. This synergism resulted in a 7-fold reduction in the total amount of cellulase loading required to achieve a similar extent of hydrolysis [3]. As the Multifect Xylanase preparation used for this earlier work was enriched in several xylanases, we were not able to identify which protein or proteins acted synergistically with the cellulases. To try to identify which of the proteins resulted in this significant synergistic interaction, we purified the major cellulase monocomponents present in Celluclast 1.5 L and the major xylanase present in Multifect Xylanases.

**Figure 1:**
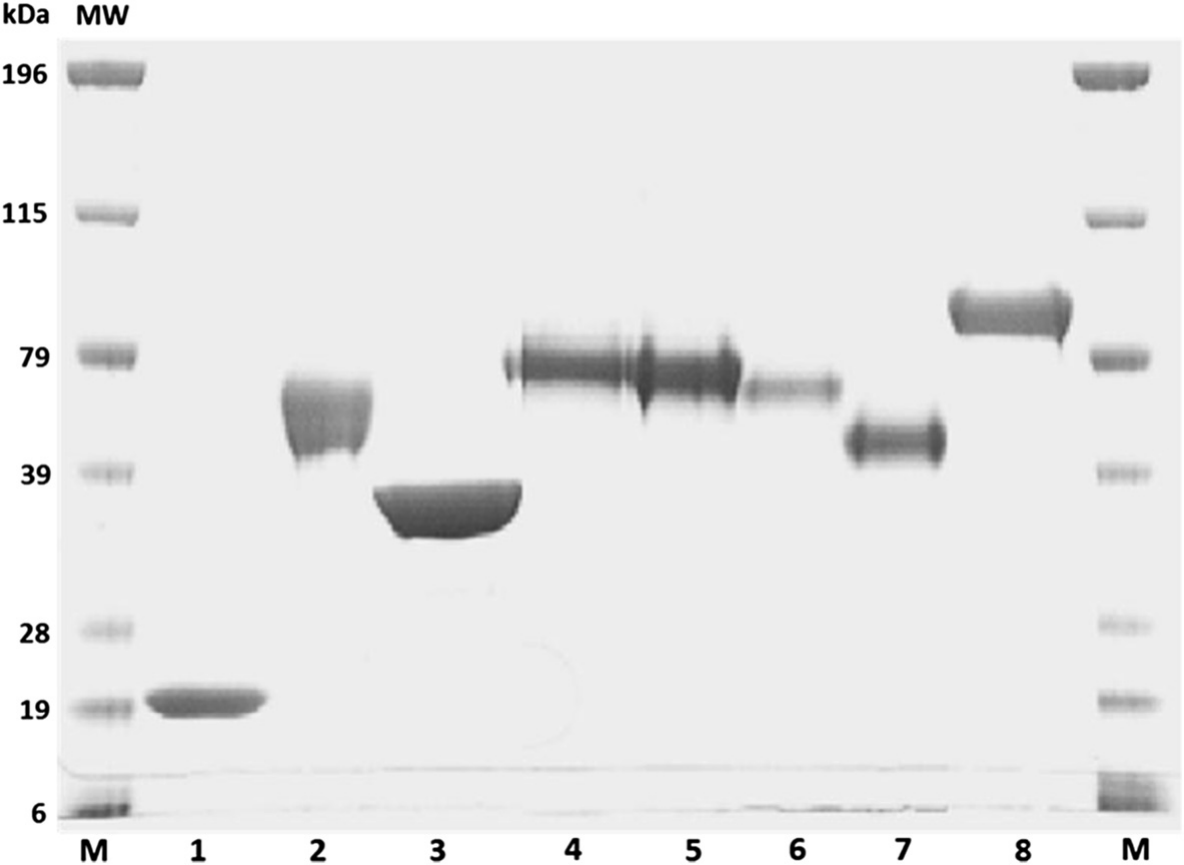
**SDS-PAGE of purified enzymes: GH11 EX (lane 1), GH10 EX (lane 2), GH5 XG (lane 3), Cel7A (lane 4), Cel6A (lane 5), Cel7B (lane 6), Cel5A (lane 7), GH3 BG (lane 8) and marker (lane M).** Proteins were identified by LC-MS/MS. Proteins are named according to their glycoside hydrolase family.

The major protein within the Multifect Xylanase was found to be GH11 EX, as shown by tandem mass spectrometry, with this protein constituting more than 80% of the total protein present in the mixture. As expected, it had a relatively low molecular weight of about 20 kDa (Figure 1). The major cellulase monocomponents within Celluclast 1.5L, on a protein weight basis, were T. *reesei* Cel7A, Cel6A, Cel7B, and Cel5A (Figure 1), which comprise about 56%, 12%, 5%, and 6% of the total protein respectively. These values were similar to the proportion of protein concentrations reported earlier by other workers [27,28].

The possible synergistic interaction between the GH11 EX and the cellulase monocomponents, Cel7A, Cel6A, and Cel5A was first assessed using both a “model” cellulosic substrate (dissolving pulp, DP) and a pretreated biomass substrate (SPCS). As Cel7B had previously been shown to exhibit significant levels of endo-xylanase activity [29,30], its interaction with GH11 EX was not assessed. When dissolving pulp was used as the substrate, the addition of GH11 EX enhanced the hydrolytic activity of both of the exo-type cellulases (Cel7A and Cel6A) but not the endo-type cellulase (Cel7B), as determined by the amount of glucose released (Table 3). When the enzyme interactions were assessed on the SPCS substrate, the addition of GH11 EX enhanced the hydrolytic potential of all of the cellulase monocomponents (Cel7A, Cel6A, and Cel5A). In all cases, the highest enhanced cellulolytic activity promoted by GH11 EX was observed with Cel7A (Table 3). Although the BSA protein control, used to substitute for GH11 EX, also improved the hydrolytic activity of the cellulase enzymes (Table 3), the slight increase in hydrolysis due to BSA addition was substantially lower than the benefit observed after GH11 EX addition. This beneficial action combined with the previous observation that GH11EX alone showed no hydrolytic activity towards either the DP or SPCS substrates indicated that the greater hydrolytic potential exhibited by the cellulase monocomponents after GH11 EX addition was the result of the synergistic interaction of the GH11 xylanase and cellulase monocomponents. As Cel7A displayed the highest degree of synergism with GH11 EX on both the DP and SPCS substrates, it was used to try to better define the mechanisms behind the observed synergistic interaction between the enzymes.

**Table 3:** Cellulose hydrolysis of dissolving pulp (DP) and steam pretreated corn stover (SPCS) by cellulase monocomponents with or without supplemental GH11 EX after 72 h

	**Cel7A**	**Cel7A + GH11 EX**	**Cel7A + BSA**	**Cel6A**	**Cel6A + GH11 EX**	**Cel6A + BSA**	**Cel5A**	**Cel5A + GH11 EX**	**Cel5A + BSA**
**DP**	41.4%	51.3%	48.4%	10.8%	12.1%	11.7%	9.6%	8.8%	8.6%
**SPCS**	22.2%	30.1%	24.9%	3.4%	5.5%	3.9%	0.5%	1.5%	0.9%

Control: BSA.

### Enhancement of the hydrolytic activity of Cel7A on various lignocellulosic substrates by the addition of accessory enzymes

**Figure 2:**
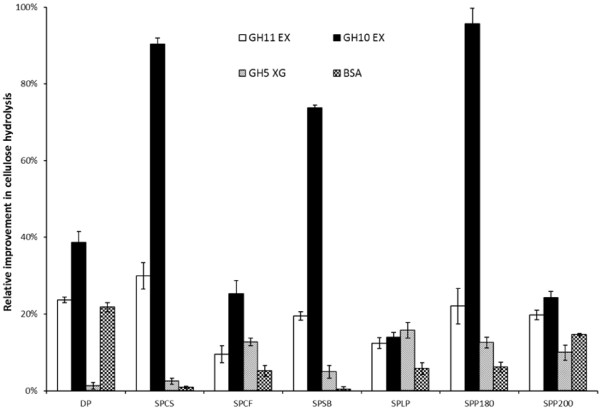
**Relative improvement in cellulose hydrolysis by supplementation of accessory enzymes (GH11 EX, GH10 EX and GH5 XG) to Cel7A during hydrolysis of various pretreated lignocellulosic substrates (SPCS, SPCF, SPSB, SPLP, SPP180 and SPP200) after 72 h.** Substrate control: dissolving pulp (DP). Protein control: BSA.

The potential of the hemicellulose-hydrolyzing enzymes GH10 EX, GH11 EX, and GH5 XG to enhance the hydrolytic activity of Cel7A was next assessed on the dissolving pulp and the various pretreated lignocellulosic substrates (Figure 2). It was apparent that both of the endo-xylanases (GH10 EX and GH11 EX) could effectively enhance the cellulolytic activity of Cel7A on all of the pretreated lignocellulosic substrates tested. Compared to GH11 EX, the addition of GH10 EX resulted in substantial increases in the hydrolytic action of Cel7A. The range of improvement varied from 10 to 100% depending on the substrate that was used. A greater increase in hydrolysis due to GH10 EX addition was observed with the relatively higher xylan containing substrates such as SPCS (7.0%), SPSB (9.8%) and SPP180 (6.6%), resulting in ≥ 80% increase in the catalytic activity of the supplemented Cel7A (Figure 2). However, there was only a modest increase in the hydrolytic activity of Cel7A when each of the endo-xylanases was supplemented during the hydrolysis of the SPCF substrate, despite its high xylan (15%) content (Table 1). Endo-xylanases also significantly improved the hydrolytic activity of Cel7A when added to substrates containing very low or virtually no xylan such as the DP and SPLP substrates (Table 2). Although the addition of the BSA controls resulted in improvements in the range of 0.5-20% (Figure 2), these increases in the hydrolytic potential of Cel7A were again, substantially lower than those achieved after GH10 EX and GH11 EX supplementation.

We next used the two steam pretreated poplar samples with differing hemicellulose content (SPP180 (6.6% xylan) and SPP200 (3.7% xylan)) to assess the possible influence of the xylan on the hemicellulose-depolymerizing enzyme’s ability to improve the cellulolytic activity of Cel7A. The addition of the GH11 EX resulted in similar hydrolysis improvements (about 20%) on both substrates despite the almost 2-fold higher xylan content in the SPP180 substrate as compared to the SPP200 substrate (Table 2). In contrast, GH10 EX addition improved the Cel7A catalytic activity 4-fold when applied to the SPP180 substrate (95%) compared to the hydrolysis yield observed with the SPP200 substrate (24%).

The trends observed in Figure 2 showed that GH5 XG addition slightly improved Cel7A activity when acting on the SPCF and SPP180 substrates whereas a significant improvement (about 20%) was observed on the SPLP substrate. It appears that the greatest improvements in Cel7A hydrolytic activity were observed when the supplemental enzymes were added to the hydrolysis of the pretreated wood substrates (SPP180, SPP200, and SPLP).

To try to assess the possible synergistic cooperation between cellulases and hemicellulases as a function of hemicellulases loading, the SPCS was hydrolyzed with cellulases supplemented with varying amounts of GH10 EX. The titration curve indicated that the cellulose hydrolysis boosting effect of adding xylanases decreased slightly (from ~90% to ~85%) when GH10 EX loading was decreased by half (5 mg/g glucan). Further decreasing the GH10 EX enzyme loading to 3 and 1 mg/g glucan could still improve cellulose hydrolysis by ~50% and ~18%, respectively.

### Enhancement of the hydrolytic activity of Cel7A on various lignocellulosic substrates by the addition of binary mixtures of accessory enzymes

**Figure 3:**
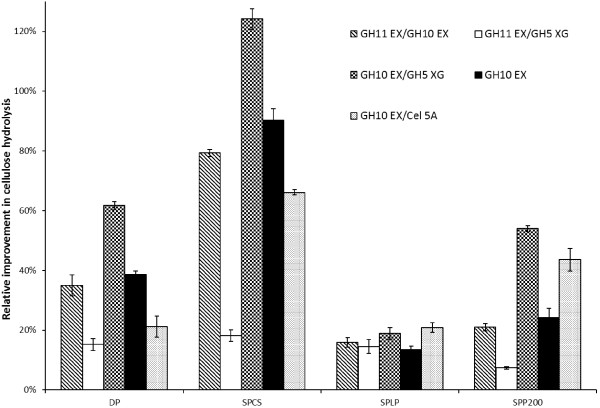
**Relative improvement in cellulose hydrolysis by supplementation of binary mixtures of accessory enzymes (GH11EX/GH10EX, GH11EX/GH5XG and GH10EX/GH5XG) to Cel7A during the hydrolysis of various pretreated lignocellulosic substrates (SPCS, SPLP and SPP200) at 72 h.** Substrate control: dissolving pulp (DP). Enzyme control: GH10EX and GH10EX/Cel5A.

To assess the possible synergistic interaction between the accessory enzymes and Cel7A, binary mixtures of GH10 EX/GH11 EX, GH10 EX/GH5 XG, and GH11 EX/GH5 XG were formulated and compared with the hydrolysis obtained when Cel7A was supplemented with GH10 EX alone (Figure 3). With the exception of the binary mixture GH11 EX/GH5 XG, all of the other binary mixtures resulted in a similar or an increase in hydrolytic activity as compared to when Cel7A was supplemented with GH10 EX alone. The greatest improvement was observed with binary mixture GH10 EX/GH5 XG, which enhanced the glucose yields obtained from the SPCS, SPP200, DP and SPLP substrates by 120, 60, 60 and 20% respectively (Figure 3). As GH5 XG had previously exhibited detectable CMCase activity (Table 2), we next substituted GH5 XG in the binary mixture GH10 EX/GH5 XG with a purified endo-glucanase (Cel5A) to assess the interaction of GH10 EX with a “true” endoglucanase enzyme. Although the addition of the GH10 EX/Cel5A mixture resulted in the improved hydrolytic activity of the Cel7A on the SPLP and SPP200 substrates as compared to the addition of GH 10 EX alone, these improvements were similar (on SPLP) or lower (on DP, SPCS and SPP200) to those observed with the GH10 EX/GH5 XG mixture. This suggested that the detected endo-glucanase activity of GH5 XG did not explain the strong synergistic interaction observed with the binary mixture GH10 EX/GH5 XG.

### Subsequent GH10 EX addition releases bound Cel7A and restarts hydrolysis after glucose yields have levelled off

**Figure 4:**
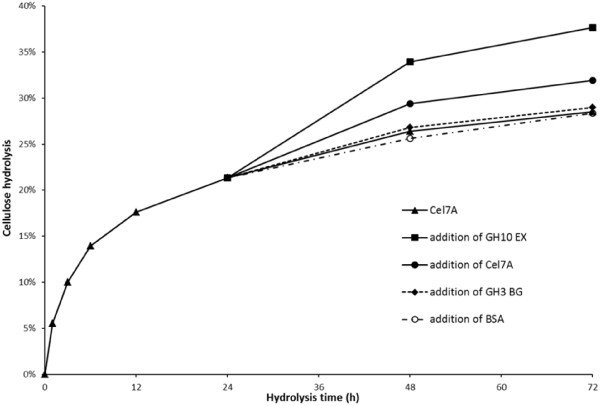
Time course of hydrolysis of steam pretreated corn stover (SPCS) by (▲) 15 mg Cel7A with the addition of (■) 10 mg GH10 EX, (●) 10 mg Cel7A, (♦) 10 mg GH3 BG and (○) 10mg BSA at 24 h.

To try to further determine the possible mechanisms involved in the observed improvements in the cellulolytic activity of Cel7A due to GH10 EX addition, hydrolysis experiments were carried out where the SPCS substrate was initially hydrolyzed with Cel7A followed by addition of GH10 EX (or other protein control) at a time when cellulose hydrolysis was observed to start levelling off. The hydrolysis of SPCS by Cel7A followed a typical hydrolysis profile (as shown in Figure 4) where, after an initial rapid rate of hydrolysis, the rate gradually decreased. The addition of more Cel7A after 24 h to the prehydrolyzed substrate resulted in an increase in hydrolysis, from 21 to 28% after 48 h, and from 28 to 32% after 72 h. However, the addition of GH10 EX rather than the Cel7A to the 24 h-prehydrolyzed SPCS resulted in significantly better cellulose hydrolysis, with the yields increased from 21 to 33% after 48 h and from 33 to 38% after 72 h (Figure 4). Controls where BSA and GH3 BG were each added after 24 h to the prehydrolyzed substrate did not promote a significant increase in cellulose hydrolysis.

**Figure 5:**
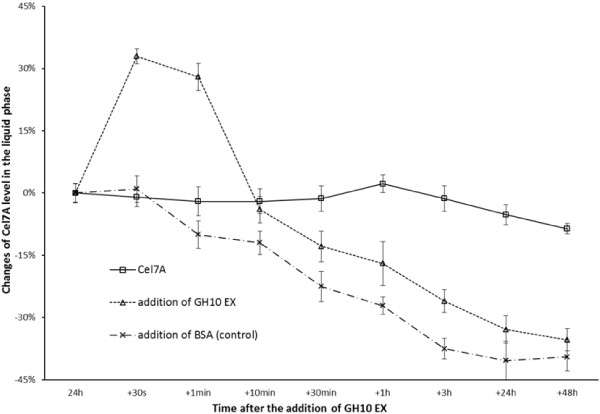
**Change of Cel7A level in the liquid phase of steam pretreated corn stover SPCS after 24 h hydrolysis (∆) with and (□) without addition of GH10 EX.** Control: (_*_) addition of BSA.

We next tried to quantify the adsorption/desorption profile of Cel7A after 24 h hydrolysis in the presence and absence of GH10 EX or the protein control (BSA) (Figure 5). In general, when Cel7A alone was added, its adsorption profile did not change during the following 48 h, other than a slight decrease in the amount of protein detected in the supernatant after this additional 48 h. However, the addition of GH10 EX resulted in a different Cel7A adsorption/desorption profile with almost 40% of the Cel7A desorbed within 60 seconds after the addition of GH10 EX. After a further 10 min, the amount of Cel7A in solution started to gradually decrease, indicating the re-adsorption of the Cel7A onto the SPCS substrate.

## Discussion

It is recognised that one of the ongoing challenges for any biomass-to-sugars process is to use minimum protein loadings to achieve good sugar yields on heterogeneous lignocellulosic substrates. Recent work [3,4] has shown how important the role that so-called accessory enzymes (xylanases, AA9, etc.) can play in achieving this goal. Although all of the xylanases assessed in the current work acted synergistically with cellulases to enhance the hydrolysis of the cellulose present in a range of pretreated lignocellulosic substrates, the extent of improvement was highly dependent on the substrate used as well as on the nature of the accessory enzyme added. In general, the endo-xylanases interacted synergistically with the cellulases in the hydrolysis of the steam pretreated agriculture residues (SPCS, SPSB) and hardwood (SPP180, SPP200). These substrates had a relatively high xylan content. In contrast, as was previously observed by Benko et al., [20], the greatest hydrolysis boosting effect obtained with xyloglucanase addition was observed during hydrolysis of steam pretreated softwood (SPLP), which contained virtually no xylan (Table 1). Interestingly, despite the much higher xylan content of the SPCF substrate (15.3%) as compared to, SPCS (7%) and SPSB (9.8%), no significant enhancement in cellulose hydrolysis was observed after endo-xylanases addition (Figure 2). This could have been due to the highly branched xylan structure of the SPCF substrate which would be expected to restrict the accessibility of endo-xylanase towards the glycosidic bonds within the xylan backbone [31,32].

Previous work has suggested that GH11 EX can act as an important accessory enzyme capable of boosting biomass hydrolysis due to its ability to effectively hydrolyze various xylanolytic substrates [10,33]. Its relatively small size is also thought to facilitate access to the xylan backbone in the complex cellulose-hemicellulose-lignin matrix [9,34]. However, among the hemicellulases that were assessed, despite the higher xylanase activity of GH11 EX when compared to GH10 EX, the GH10 EX resulted in a greater synergistic interaction than did the GH11 EX during the hydrolysis of all of the pretreated lignocellulosic substrates. This observation agrees with previous work [35] which suggested that “model” substrates used to detect enzyme activity do not necessarily predict their hydrolytic performance on heterogeneous lignocellulosic materials. The greater boosting effect of adding supplemental GH10 EX to cellulases was likely due to its ability to extensively cleave xylan to smaller chain products [17,36]. It has long been suggested that, the longer the xylo-oligosaccharides, the greater the extent of cellulases inhibition [37,38]. In addition, GH10 EX has also been shown to have a higher affinity to the highly branched xylan backbone [36,39] and to be more tolerant than GH11 EX to plant protein inhibitors such as TAXI and XIP [40].

Although earlier work [19] had shown cooperative interaction between accessory enzymes such as GH11 EX and GH 10 EX to enhance glucose release during lignocellulose hydrolysis, we found that the binary mixtures of these two family endo-xylanases mixture (GH11 EX/GH10 EX) resulted in equal or slightly lower improvements in cellulose hydrolysis yields than did the addition of GH10 EX alone to the cellulases. However, this earlier work [19] used AFEX pretreated corn stover, in contrast to the SO_2_-catalyzed steam explosion pretreatment used in this study. It is likely that the AFEX treatment left a far higher amount of residual hemicellulose in association with the cellulose, likely requiring the cooperation of both family endo-xylanases to achieve effective cellulose hydrolysis.

Although the GH11 EX/GH5 XG mixture did not appear to offer any advantages in enhancing the hydrolytic activity of Cel7A beyond the enhancement observed with the individual enzymes, a strong synergistic cooperation was observed with the GH10 EX/GH5 XG mixture. Although the exact mechanisms behind this cooperation have yet to be fully resolved, it is likely that accessory enzymes with broader substrate specificities (such as GH10 EX and GH5 XG) result in stronger cooperative interactions with cellulase enzymes and therefore the greater hydrolysis yields observed with the pretreated biomass substrates.

One of the ways that accessory enzymes are thought to aid in achieving better hydrolysis is by helping release or dislodge cellulases that seem to be stuck on the substrate. For example, Eriksson et al. [41] observed a change in the adsorption equilibrium of Cel7A upon addition of Cel7B when the hydrolysis of steam pretreated spruce had levelled off. They suggested this was due to competition between the two cellulases. It is likely that, in the work reported here, the observed change in the adsorption equilibrium of Cel7A upon addition of GH10 EX is also due to competitive adsorption. It is also worth noting that, like Cel7B, GH10 EX has a cellulose binding module with high affinity to cellulose [42,43]. Previous work has shown that Cel7A gets stuck on cellulose microfibrils during enzymatic cellulose hydrolysis [44], leading to a substantial decrease in the rate of hydrolysis. It is possible that the GH10 EX hydrolyzed possible obstacles such as xylan that restricted the processive movement of the Cel7A.

One of the main challenges in achieving effective cellulose hydrolysis is to overcome the still-not-well-understood gradual decrease in hydrolysis rate in the latter stages of hydrolysis. Various mechanisms are thought to play a role such as the inactivation of cellulase enzymes (denaturation, product inhibition, unproductive binding) and an increase in the recalcitrance of the residual substrate [41,45]. The results presented here indicate that two of the major roles that accessory enzymes such as xylanases might play in enhancing the hydrolytic potential of a “cellulase mixture” is to both help release cellulases that are stuck on the substrate while hydrolysing the non-cellulose components of the substrate that restrict access of the cellulases to the cellulose.

## Methods

### Lignocellulosic substrates pretreatment and composition

Three different agricultural residues (corn stover, sweet sorghum bagasse, and corn fiber), one hardwood (poplar), and one softwood (lodgepole pine) substrates were steam pretreated as described earlier [46-48]. Pretreatment conditions were a compromise, based on the previous work to both maximise overall sugar recovery (hemicellulose and cellulose) while providing a cellulosic component that could be readily hydrolysed with relatively low enzyme loadings. A poplar sample (SPP180) was also pretreated at low severity in order to maintain a relatively high hemicellulose/xylan content in the water insoluble cellulosic fraction. The chemical composition of the water insoluble fraction after steam pretreatment was determined using the modified Klason lignin method derived from the TAPPI standard method T222 om-88, as previously described [49]. The pretreatment conditions and chemical composition of the pretreated substrates are shown in Table 1. Dissolving pulp (DP) was used as an almost pure cellulolytic substrate control (less than 0.5% lignin, less than 3% xylan).

### Enzymes purification

Four major cellulase components of T. *reesei*, Cel7A (CBHI), Cel6A (CBHII), Cel7B (EGI), and Cel5A (EGII) were purified from Celluclast 1.5 L (Novozymes, Franklington, NC) as previously reported [28,50-52]. Family 11 endo-xylanase (GH11 EX) and family 3 β-glucosidase (GH3 BG) were purified from Multifect Xylanases (Genencor US Inc., Palo Alto, CA) and Novozyme 188 (Novozymes A/S, Bagsvaerd, Denmark), respectively, as described by [53]. In brief, family 10 endo-xylanase (GH10 EX) was purified from H-Tec (Novozymes, Franklington, NC) through two steps of chromatography. First the enzyme mixture was injected into a size exclusion column (Hiload 16/60 Superdex 75 prep grade column) with an isocratic flow of triethanol amine (TEA) buffer (20 mM, pH 7.0). The peaks containing the majority of the endo-xylanase activity were collected and further purified by ion exchange chromatography (using an ion exchange UNO Q1 column) with a linear gradient change of buffer stock A (20 mM TEA, pH 7.0) to buffer stock B (20 mM TEA, 1 M Nacl, pH 7.0). The family 5 xyloglucanase (GH5 XG) derived from *Paenibacillus sp*. was purchased from Megazyme and desalted using a sodium acetate buffer (50 mM, pH 4.8).

All purification procedures were performed in an automated FPLC system (BioLogic Due-Flow). The buffers used for enzyme purification were prepared using nanopure water and filtered through a 0.22 μm membrane filter (Millipore) followed by sonication for at least 30 min. The purity of the enzymes and lack of contamination by other cellulases and hemicellulases was confirmed by SDS-PAGE and Liquid chromatography–mass spectrometry/mass spectrometry (LCMS/MS) as described by Pribowo et al., [54].

### Enzymatic hydrolysis

The purified enzymes were used in different combinations and the cellulase monocomponents (Cel7A, Cel6A, Cel7B, and Cel5A) were assessed at a loading of 15 mg/g cellulose while the accessory enzymes (GH11 EX, GH10 EX, and GH5 XG) were individually assessed at loading of 10 mg/g cellulose, or at a loading of 5 mg/g cellulose when used in a binary combination. All of the reconstituted enzyme mixtures were supplemented with GH3 B-glucosidase at a loading of 2.5 mg/g cellulose.

The hydrolysis assays were carried out at 2% (w/v) solids loading in sodium acetate buffer (50 mM, pH 4.8) in an 8 ml total volume. The reaction mixtures were mechanically shaken in an orbital shaker incubator (Combi-D24 hybridization incubator) at 50°C for up to 72 h. The restart hydrolysis experiments were carried out by incubating steam pretreated corn stover (SPCS) with Cel7A for 24 h. Thereafter, accessory enzymes and different protein controls were added to the pre-hydrolyzed mixture and incubated for a further 48 h.

The hydrolysis was terminated by boiling the reaction mixture at 100°C for 10 min to inactivate the enzymes. The supernatants collected after centrifugation at 16000 g for 10 min and stored at −20°C for further analyses. Substrate and enzyme blanks were run at the same time by incubating the substrates without enzymes and by incubating the enzymes without substrates, respectively.

### Analytical methods

The specific activities of the purified enzymes are detailed in Table 2. The xylanase, xyloglucanase, and carboxymethyl cellulose activities (CMCase) were assessed as described by [55]. The cellobiohydrolase, β-glucosidase and β-xylosidase activities were determined using *p*-nitrophenyl-β-D-cellobioside (*p*-NPC), *p*-nitrophenyl-β-D-glucopyranoside (*p*-NPG), and *p*-nitrophenyl-β-D-xylopyranoside (*p*-NPX) as substrates, respectively, according to [56]. The protein content was measured by the Ninhydrin assay using bovine serum albumin (BSA) as the protein standard [57].

In the restart hydrolysis experiments, the adsorption/desorption profile of Cel7A after the addition of various enzymes and BSA was determined using an immunoassay to specifically quantify the amount of Cel7A present in the supernatant. Briefly, a monoclonal antibody (MAb) specific for Cel7A was used to distinguish this enzyme from the other enzymes present in the supernatant. A Cel7A polyclonal antibody (PAb) was then used to bind the captured Cel7A. The amount of Cel7A was indirectly quantified by measuring the bound PAbs using a third antibody conjugated to alkaline phosphatase (AP, Biorad). The quantitation was achieved by adding p-nitrophenylphosphate (Bio-Rad), a substrate for alkaline phosphate, and the reaction was incubated at room temperature for 30 min. The reaction was stopped by adding 400 mM glycine-NaOH. The amount of Cel7A was then indirectly quantified by measuring the absorbance of p-nitrophenyl at 405 nm.

The quantitative analysis of chemical compositions of various steam pretreated lignocellulosic substrates after Klason procedure were performed by high performance anion exchange chromatography (Dionex DX-3000, Sunnyvale, CA) as described earlier [58]. The quantitative analysis of glucose concentration in the hydrolysate was performed by Glucose Oxidase Assay [59]. The extent of cellulose hydrolysis of the pretreated substrates was calculated as a percentage of the theoretical glucan available in the substrate. All hydrolysis experiments were performed in duplicate and the mean values and standard deviations are presented.

## Abbreviations

SPCS: Steam pretreated corn stover; SPCF: Corn fiber; SPSB: Sweet sorghum bagasse; SPLP: Lodgepole pine; SPP180: Poplar steam pretreated at 180°C; SPP200: Poplar steam pretreated at 200°C; DP: Dissolving pulp; BSA: Bovine serum albumin; GH11 EX: Glycoside hydrolase family 11 endo-xylanase; GH10 EX: Family 10 endo-xylanase; GH5 XG: Family 5 xyloglucanase; GH3 BG: Family 3 β-glucosidases.

## Competing interests

The authors declare that they have no competing interests.

## Authors’ contributions

All authors (JH, VA, AP and JNS) contributed jointly to all aspects of the work reported in the manuscript. All authors have read and approved the final manuscript.
